# Effect of preexisting human leukocyte antigen donor-specific antibodies especially human leukocyte antigen-DQ on kidney transplant outcome

**DOI:** 10.3389/fneph.2026.1734903

**Published:** 2026-03-12

**Authors:** Sonia Mehrotra, Raj Kumar Sharma, Rakesh Kapoor, Rajesh Kumar Jaiswal, Rohit Kapoor

**Affiliations:** 1 Department of Molecular Biology & Immunology, Medanta Super Speciality Hospital, Lucknow, Uttar Pradesh, India; 2 Department of Nephrology and Kidney Transplant Medicine, Medanta Super Speciality Hospital, Lucknow, Uttar Pradesh, India; 3 Department of Kidney Transplant and Urology Institute, Medanta Super Speciality Hospital, Lucknow, Uttar Pradesh, India

**Keywords:** antibody-mediated rejection, ATLG, donor-specific antibodies, HLA-DQ antibodies, kidney transplantation, single antigen bead assay

## Abstract

**Background:**

Anti-HLA-DQ donor-specific antibodies are increasingly recognized for their role in early rejection and compromised graft function following kidney transplantation.

**Methods:**

A total of 119 prospective kidney transplant recipients were evaluated for pre-transplant HLA sensitization using single antigen bead (SAB) assays for class I and class II donor-specific antibodies (DSAs). All patients had negative complement-dependent cytotoxicity (CDC) crossmatch results; however, flow cytometry crossmatch was positive for T cells in three patients and showed borderline B-cell positivity in one patient. Of these patients, 100 proceeded to kidney transplantation, including 19 ABO-incompatible transplants. All recipients were followed for a minimum of 4 years post-transplant, and induction immunosuppression was administered using either anti-thymocyte globulin (ATG) or ATLG (Grafalon^®^).

**Results:**

A total of 100 patients underwent kidney transplant. Among them, 34 recipients (34%) had class I HLA antibodies (MFI range: 9,057 to 757) and 5 had class I DSAs (MFI range: 2,084 to 822) without any rejection episodes. Thirty-eight patients (38%) tested positive for class II HLA antibodies, including 20 with anti-HLA-DQ (MFI range: 7,725 to 766); of these, eight had donor-specific anti-DQ antibodies. Only one patient, who underwent an ABO-incompatible transplant and had pre-transplant DQ DSA with MFI 7,725, developed biopsy-proven antibody-mediated rejection (ABMR) but recovered following treatment. All eight DQ DSA-positive recipients showed post-transplant MFI decline within 1 month. Rejection was notably infrequent in recipients who received Grafalon^®^ induction.

**Conclusions:**

Preformed anti-HLA-DQ DSAs, especially with MFI >5,000 and in the context of ABO incompatibility, may predispose to AMR. DQ DSAs with lower MFI require vigilant monitoring due to the risk of post-transplant rebound. ATLG-based induction was associated with low rejection incidence and favorable short-term outcomes.

## 1 Introduction

Kidney transplantation remains the gold-standard treatment for patients with end-stage renal disease (ESRD), offering improved survival, quality of life, and cost-effectiveness compared to long-term dialysis (1). However, long-term graft survival is threatened by immune-mediated complications, most notably antibody-mediated rejection (AMR), which is strongly associated with donor-specific anti-human leukocyte antigen (HLA) antibodies (DSAs) (2). DSAs may arise due to prior sensitization events such as pregnancy, blood transfusion, or previous transplants, or may develop *de novo* after transplantation (3). Among the various HLA loci, antibodies targeting HLA-DQ antigens have emerged as clinically significant due to their high immunogenicity, prolonged persistence in circulation, and strong association with chronic allograft injury (4, 5).

Recent epidemiological data have shown the increasing recognition of HLA-DQ antibodies as key mediators in transplant immunobiology (6). Studies from single- and multicenter cohorts suggest that anti-DQ donor-specific antibodies (DSAs) constitute up to 55%–70% of all *de novo* DSAs detected post-transplant. These antibodies are more frequently implicated in biopsy-proven AMR, transplant glomerulopathy, and long-term graft dysfunction compared to DSAs against HLA-A, HLA-B, or HLA-DR antigens (7). Despite advances in histocompatibility testing and desensitization strategies, class II DSAs—particularly anti-HLA-DQ—remain a major therapeutic challenge (8, 9). These antibodies often persist in circulation; are relatively resistant to conventional therapies such as plasmapheresis, intravenous immunoglobulin (IVIG), and rituximab; and frequently exhibit high mean fluorescence intensity (MFI) values (10). Elevated MFI has been shown to correlate with adverse immunological outcomes. Moreover, unlike class I DSAs, class II antibodies display delayed clearance and may persist even after apparent clinical resolution of rejection episodes, further complicating long-term management (11).

In current transplant practice, most allocation systems and risk stratification models focus on panel reactive antibody (PRA) levels, prior sensitization, and donor-specific antibody mean fluorescence intensity (DSA MFI) thresholds but do not always distinguish between DSA classes or specificities. As such, clinical protocols may inadequately address the unique risks posed by HLA-DQ mismatches and antibodies. This gap is particularly concerning given accumulating evidence that anti-DQ DSAs, both preformed and arising *de novo*, are independently associated with inferior graft survival. The present study was therefore undertaken to evaluate the clinical impact of pre-transplant DSA class and specificity, with a focused analysis on anti-HLA-DQ antibodies, in a real-world cohort of live donor kidney transplant recipients.

## 2 Methodology

### 2.1 Study design

This was a prospective, observational cohort study conducted at Medanta Kidney and Urology Institute, Lucknow, a tertiary referral center for renal transplantation, between January 2020 and December 2022.

### 2.2 Aim

This study was conducted in order to evaluate the impact of preexisting donor-specific anti-HLA antibodies, with a special focus on anti-HLA-DQ antibodies, on clinical outcomes in kidney transplant recipients, particularly in the setting of ABO-incompatible transplants and under standardized desensitization and induction immunosuppression protocols.

### 2.3 Study population

Pre-transplant HLA sensitization screening was conducted in 119 prospective kidney transplant recipients. Live donor kidney transplantation was performed in 100 patients, who were included in the final analysis and followed for a minimum of 4 years post-transplant. Among these patients, 19 (19%) underwent ABO-incompatible (ABOi) transplants.

### 2.4 Study procedure

All recipients underwent panel reactive antibody (PRA) screening and single antigen bead (SAB) assay using the LIFECODES® Single Antigen Assays (LSA) by Immucor-Werfen to detect IgG class I and class II anti-HLA antibodies, including anti-HLA-DQ. DSA positivity was defined as an MFI greater than 1,000. Throughout the manuscript, MFI ranges are uniformly reported from higher to lower values to reflect the maximum observed antibody strength. Complement-dependent cytotoxicity crossmatch (CDCXM) was performed in all patients and yielded negative results in every recipient. Flow cytometric crossmatch (FCXM) was positive for T cells in three cases and showed borderline reactivity for B cells in one case. Patients undergoing ABO-incompatible or HLA-incompatible transplantation received desensitization therapy according to institutional protocol. For ABO-incompatible transplants, desensitization was initiated with alternate-day plasmapheresis or immunoadsorption and continued until anti-A or anti-B isoagglutinin titers were reduced to ≤1:8, with titers monitored before each session and on the day of transplant. In HLA-incompatible transplants, alternate-day double filtration plasmapheresis (DFPP) was employed to reduce DSA MFI levels below 1,000. In both groups, intravenous rituximab (200 mg) was administered 2 weeks prior to the scheduled transplant date as preconditioning.

### 2.5 Induction regimen

All recipients received standardized immunosuppressive therapy according to institutional protocols. For induction, patients were administered either anti-thymocyte globulin (ATG; Thymoglobulin^®^) at a dose of 1.5 mg/kg intravenously on postoperative day (POD) 0 and POD 2, or anti-human T-lymphocyte immunoglobulin (Grafalon^®^) at 3.0 mg/kg intravenously on the same schedule, based on clinician discretion. Intraoperatively, all patients received methylprednisolone 500 mg intravenously, followed by hydrocortisone 100 mg intravenously every 8 h beginning on POD 0. Maintenance immunosuppression included oral tacrolimus initiated at 0.05 mg/kg/day following pre-transplant rituximab administration and continued postoperatively (**Table 1**).

**Table 1:** The desensitization protocol.

ABO-incompatible renal transplant (ABOiRT)	
Induction therapy	IV rituximab 200 mg 2 weeks prior to the intended date of transplant.
Maintenance therapy	After a week of receiving rituximab, oral tacrolimus was started at 0.05 mg/kg/day in two equal divided doses and MMF-S at 720 mg twice daily.
Desensitization therapy	Plasmapheresis or immunoadsorption was started at this point. Alternate-day cascade plasmapheresis or immunoadsorption was continued until an anti-blood group antibody titer of 1:8 was achieved. Induction immunosuppression ATG or ATLG.
	HLA-incompatible transplant consisted of alternate-day sessions of double filtration plasmapheresis to achieve DSA MFI <1,000.
Anti-rejection therapy for graft rejection	
Antibody-mediated rejection	Methylprednisolone 500 mg × 3 doses. Plasmapheresis 5–7 sessions + IVIG 500 mg/kg body weight.
Acute cellular rejection	Methylprednisolone 500 mg × 3 doses. Antithymoglobulin, 1.5 mg/kg × 7–14 days.

### 2.6 Immunosuppression regimen

Mycophenolate sodium (MMF-S) was started at 360 mg twice daily during the desensitization phase and escalated to 720 mg twice daily after transplantation. Prednisolone tapering was performed postoperatively in accordance with institutional guidelines. Patients were followed longitudinally for up to 48 months post-transplant. Primary outcome analyses were performed at 24 months, while extended follow-up up to 48 months was used to capture late rejection episodes and long-term graft outcomes. The primary outcome was the incidence of biopsy-proven acute rejection (BPAR), classified according to the Banff 2019 criteria. Secondary outcomes included graft function [estimated glomerular filtration rate (eGFR) using the Modification of Diet in Renal Disease (MDRD) formula], graft survival, delayed graft function (DGF), patient survival, and infectious complications.

### 2.7 Statistical analysis

Descriptive statistics were used to summarize demographic and clinical characteristics. Continuous variables were expressed as mean ± standard deviation (SD), and categorical variables were presented as frequencies and percentages. Comparisons between groups (e.g., DSA-positive vs. DSA-negative; ABO-compatible vs. ABO-incompatible) were performed using the chi-square test for categorical variables and the Student’s *t*-test or the Mann–Whitney *U* test for continuous variables, as appropriate. A *p*-value of <0.05 was considered statistically significant. Analyses were performed using SPSS version 27.0 (IBM Corp., Armonk, NY, USA).

### 2.8 Ethical considerations

The study received approval from the Institutional Ethics Committee of Medanta, Lucknow (ECR/1529/Inst/UP/2021), and was conducted in accordance with the ICH-E6 GCP guidelines.

## 3 Results

### 3.1 Demographic profile

A total of 119 live donor kidney transplants were performed during the study period. The mean age of the recipients was 35.55 ± 11.27 years, and the majority were men (91.6%). Donors were predominantly women (79.8%) with a mean age of 44.28 ± 12.6 years. The mean donor GFR was 99.66 ± 17.0 mL/min/1.73 m². Detailed demographic characteristics of the patients are presented in **Table 2**.

**Table 2:** Demographic details.

Characteristics (*N* = 119)	Mean, *n* (%)
Age (recipients) (mean ± SD)	35.55 ± 11.27 (15–64 years)
Gender (recipients)	
Male	109 (91.6%)
Female	10 (8.4%)
Basic disease CKD with DM	21 (17.64%)
Basic disease CKD with HTN	77 (64.70%)
Basic disease CKD with IgA	5 (4.2%)
Basic disease CKD with DM and HTN	14 (11.76%)
Viral marker	
Positive	13 (10.9%)
Negative	106 (89.1%)
Transfusion history	46 (38.65%)
No transfusion history	73 (61.34%)
Serum creatinine (within a month)	1.104 ± 0.49
Blood group (recipients)	
“O” blood group	35 (29.41%)
“AB” blood group	15 (12.60%)
Age of donor	44.28 ± 12.6
Gender (donor)	
Male	24 (20.2%)
Female	95 (79.8%)
GFR donor (DTPA) (mean **±** SD)	99.66 ± 17.0
Blood group (donor)	
“O” blood group	39 (32.77%)
“AB” blood group	9 (7.56%)
HLA match (haploidentical)	52 (43.69%)
HLA mismatch	11 (9.24%)

#### 3.1.1 HLA class I antibody profile

**Figure 1:**
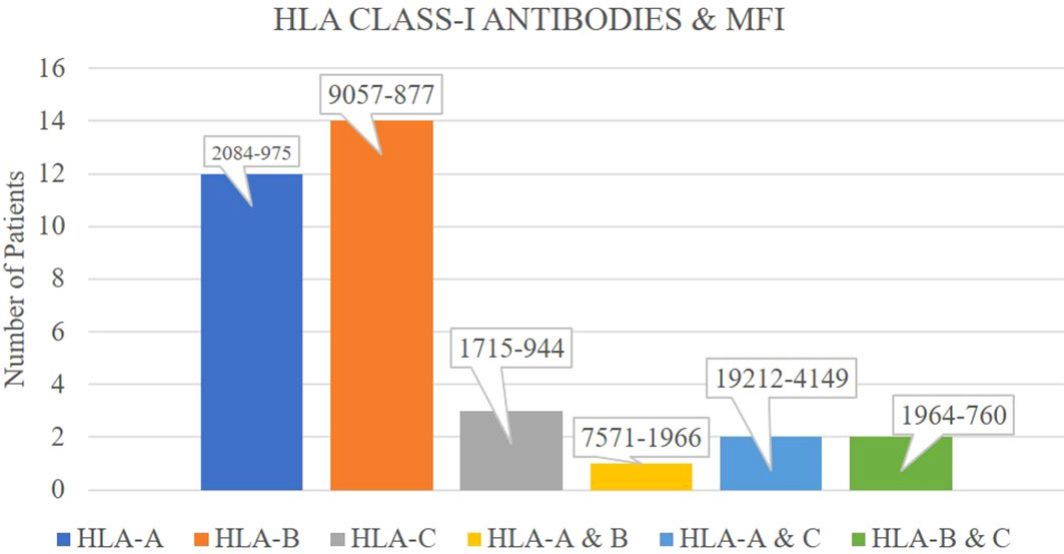
Distribution of HLA class-I antibodies among patients and their corresponding mean fluorescence intensity (MFI) values.

Among the 100 kidney transplant recipients included in the study, 34 patients (34%) were positive for HLA class I antibodies based on the SAB assay, with an MFI range of 9,057 to 757. Within this group, anti-HLA-A antibodies were detected in 15 recipients (MFI range: 2,084 to 975), anti-HLA-B in 16 recipients (MFI range: 9,057 to 877), and anti-HLA-C in 3 recipients (MFI range: 1,715 to 944). Of these 34 class I-positive individuals, only five had donor-specific antibodies (DSAs) with MFI values ranging from 2,084 to 822. Notably, none of these five recipients experienced any rejection episodes during the follow-up period (**Figure 1**).

#### 3.1.2 DSA-negative and non-specific antibody profile

**Figure 2:**
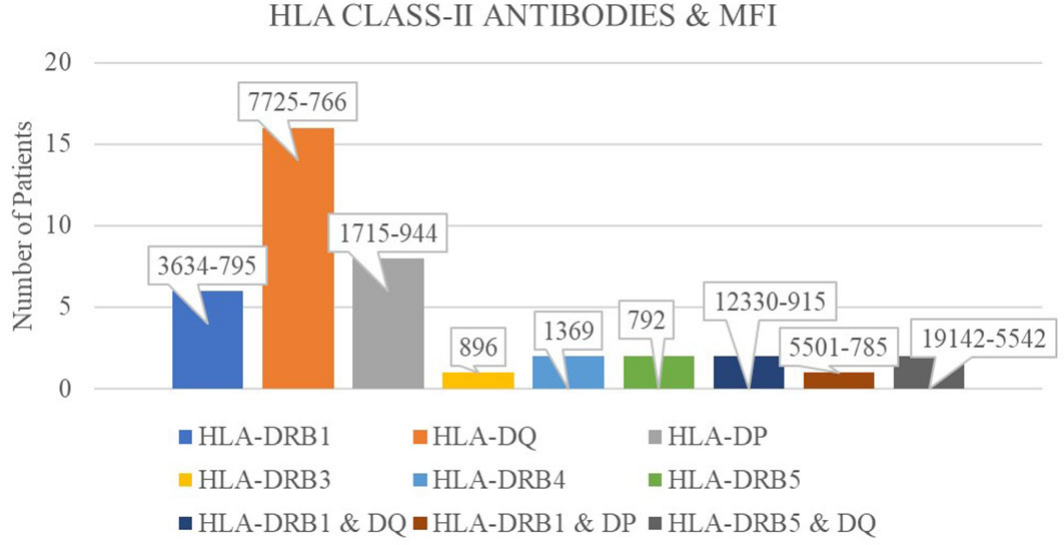
Frequency of patients exhibiting HLA class-II antibodies with their corresponding mean fluorescence intensity (MFI) values.

A total of 85 patients (85%) were negative for both class I and class II DSAs. Nine recipients (9%) had both class I and class II antibodies that were not donor specific. SAB testing further revealed that 38 recipients (38%) were positive for class II HLA antibodies. These included anti-HLA-DRB1 antibodies in 7 patients (MFI range: 3,634 to 795), anti-HLA-DQ antibodies in 20 patients (MFI range: 7,725 to 766), and anti-HLA-DP antibodies in 8 patients (MFI range: 1,715 to 944). Additionally, anti-HLA-DRB3, DRB4, and DRB5 antibodies were each identified in three recipients, with MFI values of 896, 1,369, and 792, respectively (**Figure 2**).

#### 3.1.3 Donor-specific HLA-DQ antibodies and rejection outcomes

**Figure 3:**
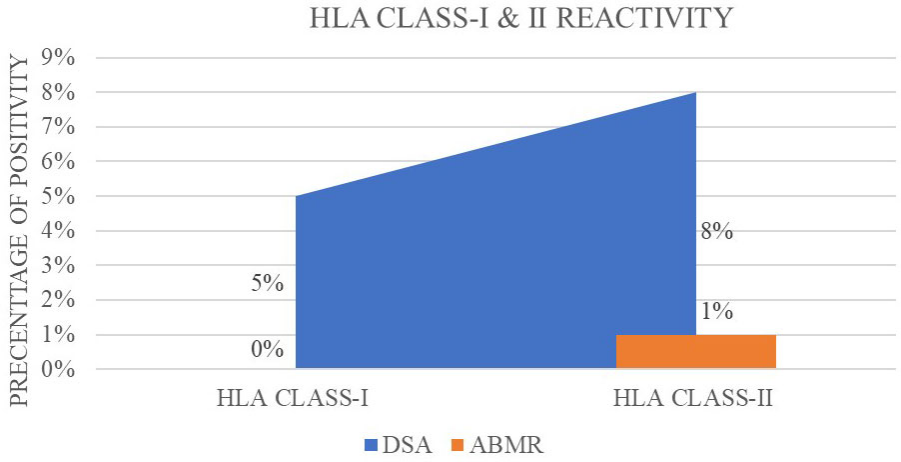
Percentage positivity of donor-specific antibodies (DSAs) and antibody-mediated rejection (ABMR) for HLA class-I and class-II reactivity.

Of the 20 recipients with anti-HLA-DQ antibodies, 8 had donor-specific HLA-DQ DSAs (MFI range: 7,725 to 776). As depicted in **Figure 3**, one of these eight patients had an ABO-incompatible transplant and developed biopsy-proven antibody-mediated rejection (ABMR) post-transplantation, with pre-transplant DSA MFI of 7,725 and post-transplant reduction to 2,555. This patient responded to treatment, and DSA MFI declined within 1 month. The remaining seven recipients with HLA-DQ DSAs did not experience rejection, and all showed a downward trend in post-transplant DSA MFI values within the first month. None of the seven remaining DQ DSA-positive recipients required intensification of maintenance immunosuppression, and no post-transplant DSA rebound was observed during the early follow-up (**Figure 3**).

#### 3.1.4 Comparison of outcomes between DSA-positive and DSA-negative recipients

At the predefined 24-month primary outcome time point, graft survival was 100% in both DSA-positive (*n* = 13) and DSA-negative (*n* = 87) groups. Importantly, none of the ABO-incompatible recipients who were DSA-negative experienced AMR; the only AMR episode occurred in an ABOi recipient with a high-MFI preformed DQ DSA. Only one biopsy-proven AMR episode occurred in an ABO-incompatible recipient with a high-MFI preformed HLA-DQ DSA (7,725). Mean serum creatinine at 2 years was comparable between DSA-positive and DSA-negative recipients (1.10 ± 0.49 vs. 1.08 ± 0.44 mg/dL, *p* = 0.78). This indicates no measurable difference in short-term graft function between the two groups. No significant differences in estimated GFR or clinical outcomes were observed. These data indicate comparable short-term and mid-term graft performance regardless of pre-transplant DSA status. At follow-up of 48 months of transplant, three recipients had late ABMR with biopsy-proven and SAB DSA positivity due to drug non-compliance.

#### 3.1.5 Induction immunosuppression and clinical correlation

Among the immunologically high-risk recipients (including ABO-incompatible and DSA-positive patients), 15 received ATLG and 4 received ATG as induction therapy. Rejection was infrequent in recipients who received ATLG; however, given that only one episode of early AMR occurred in the entire cohort, no causal inference regarding the effect of ATLG can be drawn.

## 4 Discussion

The current study was conducted to understand the prognostic role of DSA, with a focused evaluation of HLA class specificity. In our cohort, only one biopsy-proven AMR episode occurred, which inherently limits the statistical power to draw causal associations between preformed class II DSA and post-transplant outcomes. Therefore, the present findings should be interpreted as observational trends rather than confirmatory evidence. The single AMR case occurred in a recipient with a high-MFI preformed HLA-DQ DSA undergoing ABO-incompatible transplantation, suggesting a potential clinical relationship that warrants further validation in larger cohorts.

In this cohort, the predominance and pathogenicity of class II DSAs, particularly anti-DQ antibodies, are consistent with prior reports. In a multicenter retrospective analysis, Kannabhiran et al. showed that patients with persistent class II DSAs post-transplant had markedly poorer graft survival compared to those in whom DSAs were resolved, with HLA-DQ antibodies emerging as the most refractory to immunologic suppression (12). While prior studies highlight the clinical relevance of class II DSAs, particularly anti-DQ antibodies, our findings remain descriptive given the single observed AMR event. The clinical relevance of this lies in the immunobiology of HLA-DQ: its heterodimeric structure and higher immunogenic potential render it a potent stimulus for alloimmune activation. No significant difference in graft function or survival was observed between DSA-positive and DSA-negative recipients within the available follow-up period.

Reported AMR rates in ABO-incompatible kidney transplantation range from 10% to 30% in published literature, depending on the desensitization strategy (13). In our cohort, the AMR rate among ABOi recipients was 5.3% (1/19), occurring only in the presence of a high-MFI DQ DSA.

High-MFI DQ DSAs have been associated with increased rejection risk in prior literature; however, our data cannot confirm this association due to only one AMR episode. These results mirror those of Zecher et al. who reported that among various pre-transplant crossmatch modalities, only DSA MFI ≥5,000 remained independently associated with acute rejection (OR 4.14, *p* = 0.02), while CDC and FCXM positivity did not reach statistical significance (14).

The kinetics and persistence of DSAs post-transplantation also merit attention. Morrison et al. reported that weakly reactive DSAs, particularly anti-HLA-DQ, may persist after transplantation and be less responsive to standard therapies (15). In contrast, all eight recipients in our cohort with preformed DQ DSAs demonstrated an early decline in MFI within the first month, and no DSA persistence or rebound was observed during this period. These findings indicate effective short-term immunologic control in our setting, although longer follow-up would be required to assess late kinetics.

Furthermore, data from Wiebe et al. demonstrated that *de novo* DQ DSAs are highly predictive of both subclinical inflammation and chronic allograft injury (7). Although our study focused primarily on preformed DSAs, the pathophysiological implications overlap: once established, DQ-directed antibody responses are challenging to eradicate and may serve as a nidus for progressive alloimmune damage. These findings support calls for greater emphasis on HLA-DQ matching in allocation protocols, a factor still underutilized in many transplant centers globally.

The study conducted by Betjes et al. further aligns with our findings. It concluded that preformed class II DSAs are more closely linked with biopsy-proven AMR and long-term graft dysfunction than class I DSAs (16). Prior studies have shown that sustained high-MFI HLA-DQ DSAs may negatively influence long-term allograft outcomes, although our cohort did not demonstrate such changes during the follow-up period. When considered alongside our current findings, these results underscore the necessity for a redefined immunologic risk stratification model that assigns increased prognostic weight to HLA-DQ specificity.

Our study also has practical implications for post-transplant surveillance and management. The application of LIFECODES® Single Antigen Assays (LSA) with longitudinal MFI assessment serves as a clinically informative method for dynamic stratification of allograft rejection risk. The presence of HLA-DQ donor-specific antibodies warrants individualized induction immunosuppression, intensified post-transplant immunologic monitoring, and consideration of targeted therapeutic interventions aimed at B-cell or plasma cell modulation. Finally, incorporation of HLA-DQ mismatch minimization into donor–recipient allocation strategies should be prioritized, particularly in sensitized or immunologically high-risk transplant recipients.

An important practical question is whether living-donor transplantation should be deferred in the presence of preformed DSA with MFI >5,000, irrespective of antigen specificity. While our single high-risk case aligns with prior reports linking very high MFI to acute rejection, the available data are insufficient to support a blanket recommendation to avoid living-donor transplantation in all such situations. Instead, we suggest that preformed DSA with MFI >5,000 should be considered a very high-risk immunologic profile, prompting individualized decision-making, intensified desensitization, and close post-transplant surveillance.

This study has important limitations. Only one recipient developed biopsy-proven AMR, limiting the ability to perform meaningful statistical analysis regarding the impact of preformed class II DSA. Although primary analyses were performed at 24 months, extended follow-up up to 48 months may still be insufficient to fully assess late chronic antibody-mediated injury or long-term graft survival differences between DSA-positive and DSA-negative recipients. Larger multicenter studies with longer duration of follow-up are required to validate these observations.

## 5 Conclusion

In this cohort, a single early antibody-mediated rejection episode occurred in a recipient with high-MFI preformed HLA-DQ DSA who underwent ABO-incompatible transplantation. While the coexistence of high-titer DQ DSA and ABO incompatibility provides a biologically plausible context for AMR, the available data are insufficient to support a statistical association or causal inference. The observation, therefore, suggests a potential immunologic risk pattern rather than a confirmed relationship. Careful pre-transplant evaluation of DSA specificity and antibody strength, particularly for HLA-DQ, together with individualized immunosuppression and close post-transplant monitoring, remains essential. Future studies with larger sample sizes and longer follow-up are needed to clarify the true impact of preformed DQ DSA and to determine whether HLA-DQ matching should be systematically incorporated into allocation policies.

## Data Availability

The raw data supporting the conclusions of this article will be made available by the authors, without undue reservation.
