# Correction: Effect of preexisting human leukocyte antigen donor-specific antibodies especially human leukocyte antigen-DQ on kidney transplant outcome

**DOI:** 10.3389/fneph.2026.1864478

**Published:** 2026-06-30

**Authors:** Sonia Mehrotra, Raj Kumar Sharma, Rakesh Kapoor, Rajesh Kumar Jaiswal, Rohit Kapoor

**Affiliations:** 1 Department of Molecular Biology & Immunology, Medanta Super Speciality Hospital, Lucknow, Uttar Pradesh, India; 2 Department of Nephrology and Kidney Transplant Medicine, Medanta Super Speciality Hospital, Lucknow, Uttar Pradesh, India; 3 Department of Kidney Transplant and Urology Institute, Medanta Super Speciality Hospital, Lucknow, Uttar Pradesh, India

**Keywords:** antibody-mediated rejection, ATLG, donor-specific antibodies, HLA-DQ antibodies, kidney transplantation, single antigen bead assay

A correction has been made to Section: **Methodology**, 2.4 **Study Procedure**, 1:

“All recipients underwent panel reactive antibody (PRA) screening and single antigen bead (SAB) assay using the LIFECODES^®^ Single Antigen Assays (LSA) by Immucor- Werfen to detect IgG class I and class II anti-HLA antibodies, including anti-HLA-DQ”.

A correction has been made to Section: **Discussion**, 8:

“The application of LIFECODES^®^ Single Antigen Assays (LSA) with longitudinal MFI assessment serves as a clinically informative method for dynamic stratification of allograft rejection risk”.

The original version of this article has been updated.

